# Vascular Fibrosis in Aging and Hypertension: Molecular Mechanisms and Clinical Implications

**DOI:** 10.1016/j.cjca.2016.02.070

**Published:** 2016-05

**Authors:** Adam Harvey, Augusto C. Montezano, Rheure Alves Lopes, Francisco Rios, Rhian M. Touyz

**Affiliations:** Institute of Cardiovascular and Medical Sciences, BHF Glasgow Cardiovascular Research Centre, University of Glasgow, Glasgow, Scotland

## Abstract

Aging is the primary risk factor underlying hypertension and incident cardiovascular disease. With aging, the vasculature undergoes structural and functional changes characterized by endothelial dysfunction, wall thickening, reduced distensibility, and arterial stiffening. Vascular stiffness results from fibrosis and extracellular matrix (ECM) remodelling, processes that are associated with aging and are amplified by hypertension. Some recently characterized molecular mechanisms underlying these processes include increased expression and activation of matrix metalloproteinases, activation of transforming growth factor-β1/SMAD signalling, upregulation of galectin-3, and activation of proinflammatory and profibrotic signalling pathways. These events can be induced by vasoactive agents, such as angiotensin II, endothelin-1, and aldosterone, which are increased in the vasculature during aging and hypertension. Complex interplay between the “aging process” and prohypertensive factors results in accelerated vascular remodelling and fibrosis and increased arterial stiffness, which is typically observed in hypertension. Because the vascular phenotype in a young hypertensive individual resembles that of an elderly otherwise healthy individual, the notion of “early” or “premature” vascular aging is now often used to describe hypertension-associated vascular disease. We review the vascular phenotype in aging and hypertension, focusing on arterial stiffness and vascular remodelling. We also highlight the clinical implications of these processes and discuss some novel molecular mechanisms of fibrosis and ECM reorganization.

## Résumé

Le vieillissement constitue le principal facteur de risque d’apparition de l’hypertension et de la maladie cardiovasculaire. En vieillissant, le système vasculaire subit des modifications structurelles et fonctionnelles caractérisées par une dysfonction endothéliale ainsi que l’épaississement, la rigidification et la perte d’élasticité des parois vasculaires. La rigidité vasculaire est causée par la fibrose et le remodelage de la matrice extracellulaire, des processus qui sont associés au vieillissement et qui sont amplifiés en présence d’hypertension. Parmi les mécanismes moléculaires sous-jacents du vieillissement récemment identifiés, on retrouve l’augmentation de l’expression et de l’activation des métalloprotéinases matricielles, l’activation des voies de signalisation du facteur de croissance transformant bêta 1 impliquant les protéines SMAD, la régulation positive de la galectine-3 et l’activation des voies de signalisation pro-inflammatoires et profibrotiques. Ces mécanismes peuvent être induits par divers agents vasoactifs comme l’angiotensine II, l’endothéline-1 et l’aldostérone dont la présence s’accroît au fil du processus de vieillissement et en présence d’hypertension. Cette interaction complexe entre le « processus de vieillissement » et les facteurs pro-hypertensifs entraîne un remodelage et une fibrose accélérée ainsi que la rigidification des artères qu’on observe habituellement avec l’hypertension. Puisque le phénotype vasculaire de l’hypertendu jeune ressemble à celui de la personne âgée par ailleurs en bonne santé, on fait désormais de plus en plus souvent appel au vocable de vieillissement vasculaire « précoce » ou « prématuré » pour désigner la maladie vasculaire liée à l’hypertension. Nous passons ici en revue le phénotype vasculaire du vieillissement et de l’hypertension en mettant l’accent sur la rigidité artérielle et le remodelage vasculaire. Nous traitons également de l’incidence clinique de ces processus, en plus d’aborder quelques-uns des mécanismes moléculaires de la fibrose et de la réorganisation de la matrice extracellulaire.

Hypertension is the largest contributor to the global burden of cardiovascular disease. The World Health Organization estimates that the number of adults with high blood pressure will increase from 1 billion to 1.5 billion worldwide by 2020.^1^ This increase is related in part to the fact that the population is aging. Of all the factors contributing to hypertension, such as genetics, obesity, dyslipidemia, sedentary lifestyle, and diabetes, advancing age is the most important risk factor. Both aging and hypertension are associated with structural, mechanical, and functional changes in the vasculature, characterized by increased arterial stiffness, reduced elasticity, impaired distensibility, endothelial dysfunction, and increased vascular tone. The prevalence of vascular stiffness and high blood pressure increases with age and as such, hypertension has been considered to be a condition of aging. Arterial stiffening precedes the development of hypertension, and both phenomena occur more frequently in the elderly. The relationship between aging, cardiovascular disease, and vascular stiffening is further exemplified in patients with progeria (premature aging), who exhibit accelerated vascular aging and often die of cardiovascular disease.^2^ Arterial stiffening is caused primarily by excessive fibrosis and reduced elasticity, with associated increased collagen deposition, increased elastin fiber fragmentation/degeneration, laminar medial necrosis, calcification, and cross-linking of collagen molecules by advanced glycation end-products.

Fibrosis as a dynamic process initially is an adaptive repair response that is reversible. However, the fibrogenic process is progressive, leading to further worsening of arterial stiffness and fibrosis that gradually extends into the neighbouring interstitial space. Fibrosis occurs in both large and small arteries. In large vessels, vascular stiffening leads to hemodynamic damage to peripheral tissues.^3^ Fibrosis and stiffening of the resistance circulation impair endothelial function, increase vasomotor tone, promote vascular rarefaction, and alter tissue perfusion. The combination of “aging” and prohypertensive elements, such as activation of the renin-angiotensin-aldosterone system, inflammation, oxidative stress, salt consumption, and genetic factors, results in excessive arterial fibrosis and extracellular matrix (ECM) deposition with amplification of aging-related vascular injury and stiffness. These processes lead to excessive fibrosis, which often extends from small arteries and replaces parenchymal tissue, thereby leading to tissue fibrosis, scarring, and hypertension-associated target organ damage of the heart, kidney, and brain.

At the molecular and cellular levels, arterial aging and hypertension-associated vascular changes are characterized by reduced nitric oxide production, increased generation of reactive oxygen species (ROS) (oxidative stress), activation of transcription factors, induction of “aging” genes, stimulation of proinflammatory and profibrotic signalling pathways, reduced collagen turnover, calcification, vascular smooth muscle cell proliferation, and ECM remodelling. These processes contribute to increased fibrosis, which is further promoted by prohypertensive vasoactive agents, such as angiotensin II (Ang II), endothelin-1 (ET-1), and aldosterone, which stimulate profibrotic signalling cascades, including p38 mitogen-activated protein kinases (p38 MAPK) and the transforming growth factor-β (TGF-β)/SMAD pathway. Activation of galectin-3 and dysregulation of MMPs and TIMPs are involved in ECM remodelling and further enhance vascular fibrosis. Many of these events are upregulated with advancing age and in human and experimental hypertension. We review the vascular phenotype in physiological aging and in hypertension, focusing particularly on arterial stiffness and fibrosis.

## Aging-Associated Vascular Alterations

**Figure 1:**
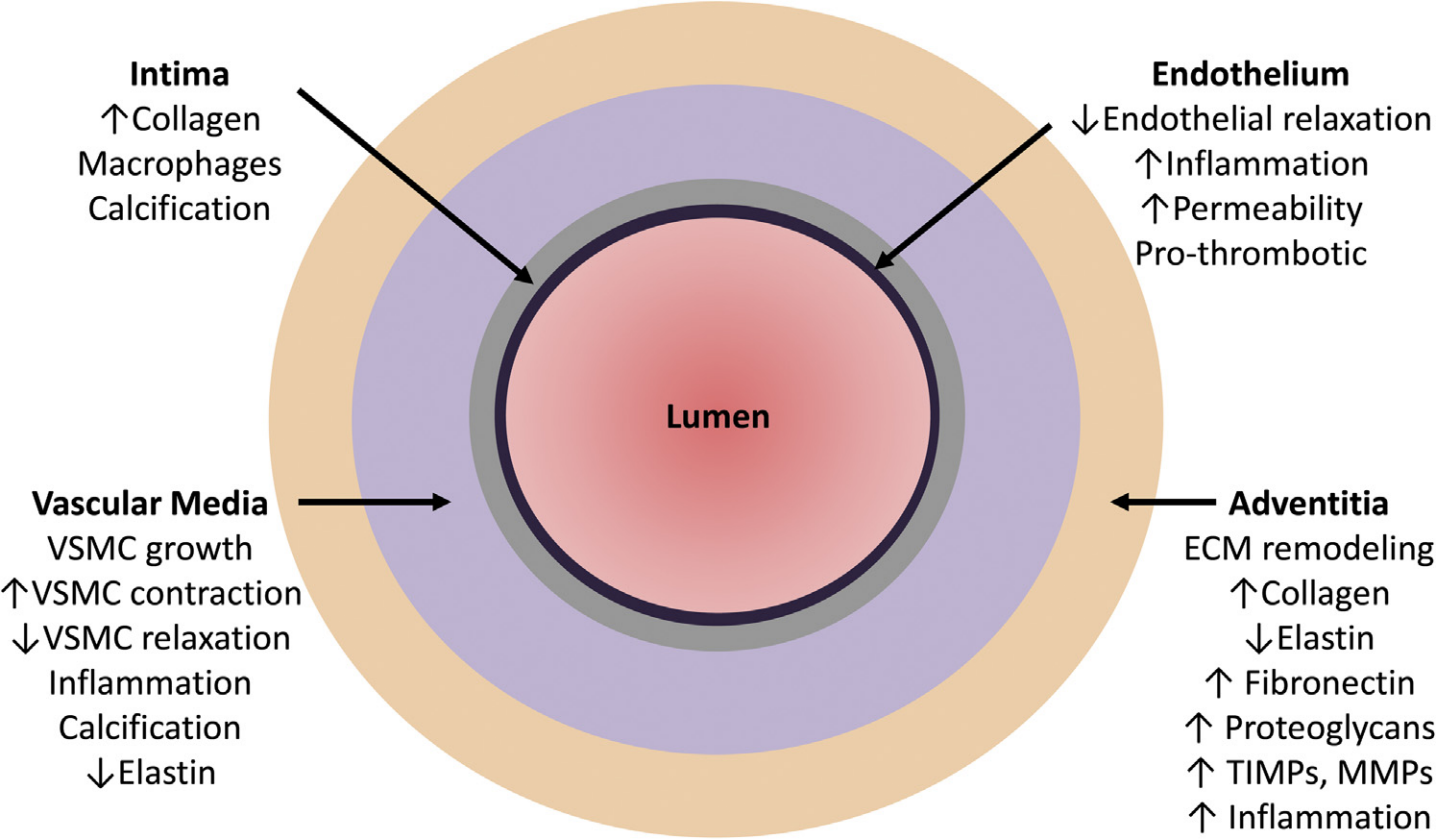
The vascular phenotype in aging and hypertension. With aging and during the development of hypertension, the endothelium, vascular wall, and adventitia undergo functional and structural changes. Endothelial function is impaired and the vascular media is thickened. The adventitial extracellular matrix undergoes remodelling, with increased collagen deposition, reduced elastin content, and increased proinflammatory cells. These processes contribute to vascular fibrosis and stiffening. ECM, extracellular matrix; MMP, matrix metalloproteinases; TIMPs, tissue inhibitory metalloproteinases; VSMC, vascular smooth muscle cell.

With aging, the vasculature undergoes functional, structural, and mechanical changes characterized by endothelial dysfunction, thickening (remodelling) of the vascular wall, and increased stiffening, respectively (Fig. 1). These changes result in a reduced capacity of arteries to adapt to tissue demands and accordingly may lead to ischemic injury. Preclinical and clinical studies have clearly demonstrated that with aging, there is impaired endothelium-dependent vasorelaxation with associated increased permeability and vascular inflammation.

Epidemiologic, cross-sectional, clinical, and postmortem studies in healthy individuals of variable ages have clearly demonstrated that intimal wall thickening and dilatation are noticeable structural changes that occur in conduit arteries with advanced age. Findings from noninvasive vascular phenotyping studies in healthy individuals have demonstrated that intima-media thickness increases 2- to 3-fold between 20 and 90 years of age.^4^ Studies in aging nonhuman primates also showed a relationship between intimal thickness in the thoracic aorta and aging.^5^ Exact factors causing progressive intimal thickening with aging in otherwise healthy individuals remain elusive, but a number of distinctive changes at the cellular and morphologic levels have been identified, including fracture of elastin fibres within the tunica media, increased collagen deposition, cellular senescence, and dysregulated cell proliferation. Associated with these events is remodelling of the ECM, which is an essential component of the connective tissue surrounding the vascular wall.

The ECM is composed of basic structural elements (collagen and elastin) and more specialized proteins including fibronectin and proteoglycans. The ECM is a dynamic structure and its components are continuously being turned over through highly regulated systems involving activation of MMPs and TIMPs. Dysregulation of these processes, together with alterations in profibrotic and proinflammatory signalling pathways, likely contribute to aging-associated vascular structural changes.

## The Vascular Phenotype in Hypertension Resembles Aging-Associated Vascular Remodelling

The overall vascular phenotype of an individual at any 1 time depends not only on “aging” but also on a combination of multiple interacting factors, such as genetic factors, diet, smoking, diabetes, dyslipidemia, oxidative stress, and obesity.^6^, ^7^ Moreover, in the presence of prohypertensive factors, there is acceleration of aging-associated vascular changes that leads to exaggerated vascular injury and arterial stiffening. In susceptible individuals, the interplay between aging and hypertension leads to “early vascular aging” and arterial stiffness, in which the vascular phenotype in young hypertensive individuals resembles that of elderly otherwise healthy individuals (Fig. 1).

## Arterial Stiffness

Normally, conduit arteries distend to accommodate large pressure ejections from the heart during systole to facilitate perfusion to tissues during diastole. This is determined in large part by the elasticity, distensibility, and compliance of the arterial system. Loss of elasticity and increased stiffness demand greater force to accommodate blood flow, leading to increased systolic blood pressure, increased cardiac work load, and consequent cardiac hypertrophy and risk of cardiovascular events. Aortic stiffness also affects the microcirculation and vice versa.^7^, ^8^ Aortic wall stiffening causes increased pulse wave velocity (PWV) and premature reflected waves with elevated central hemodynamic load leading to damage of peripheral small arteries.^9^ Remodelling of small arteries in turn leads to increased peripheral vascular and pulse wave reflection, which can further contribute to aortic stiffness.^10^ Arterial stiffness can be assessed by measuring PWV, pulse wave analysis, ambulatory arterial stiffness (using 24-hour ambulatory blood pressure monitoring) and evaluating endothelial function (flow-mediated dilation). PWV is the most commonly used approach and measures the speed of the pressure pulse from the heart as it is propagated through the arteries; it is calculated by dividing the distance travelled by the time taken to travel the defined distance. Stiffer arteries result in a more rapid travel time and hence a higher PWV. Various approaches can be used to measure PWV, including applanation tonometry, oscillometry, Doppler echocardiography, and magnetic resonance imaging. Although the measurement of PWV is considered to be the most simple, noninvasive, robust, and reproducible method to determine arterial stiffness,^11^ it is not yet used in routine clinical practice. Carotid-femoral PWV is a direct measure of aortic stiffness and is now considered the gold standard for its evaluation in clinical and epidemiologic studies.^12^

Arterial stiffness is a natural consequence of advancing age and is accelerated in hypertension. It is also an independent predictive risk factor for cardiovascular events and, as such, aortic PWV is now recognized as an important biomarker in the determination of cardiovascular risk. Arterial stiffness has a bidirectional causal relationship with blood pressure, because high blood pressure causes arterial wall injury, which promotes stiffening, whereas arterial stiffening itself is the major cause of increased systolic blood pressure, especially in the elderly,^8^, ^13^ Multiple interacting factors at the systemic (blood pressure, hemodynamics), vascular (vascular contraction/dilatation, ECM remodelling), cellular (cytoskeletal organization, inflammatory responses), and molecular (oxidative stress, intracellular signalling, mechanotransduction) levels contribute to arterial stiffness in aging and hypertension. Dysregulation of endothelial cells, vascular smooth muscle cells, and adaptive immune responses has also been implicated in arterial aging and vascular damage in hypertension. A detailed discussion of all these mechanisms is beyond the scope of this review and is addressed elsewhere this issue of the *Canadian Journal of Cardiology*.^14^ Here we focus on some molecular and cellular events that contribute to vascular fibrosis and ECM remodelling.

## The ECM and Vascular Fibrosis in Aging and Hypertension

The ECM is an essential component of the connective tissue that surrounds cells. In addition to maintaining cellular and vascular integrity, it plays a fundamental role in cell signalling and regulation of cell-cell interactions. The ECM comprises multiple structural proteins, including collagens, elastin, fibronectin, and proteoglycans. Composition of the ECM varies from organ to organ, with collagen types I and III representing the predominant isoforms in the vascular ECM.^15^ The absolute and relative quantities of collagen and elastin determine biomechanical properties of vessels, in which an elastin deficiency/collagen excess leads to vascular fibrosis and increased stiffness.^4^, ^15^ In healthy individuals, collagen deposition and turnover are tightly regulated, and the ratio of collagen to elastin remains relatively constant. However, an imbalance in these processes leads to excessive ECM protein deposition, particularly collagen and fibronectin, contributing to vascular fibrosis and stiffening in aging and during the development of hypertension.^15^ Collagens are particularly important in these processes because they are the most abundant and stiffest of the ECM proteins. Increased collagen content and destruction of the elastin fiber network together with a proinflammatory microenvironment contribute to ECM remodelling and increased intima-media thickening and vascular stiffness in small and large arteries in human and experimental hypertension.

Contributing to the profibrotic process is transglutaminase (TG2), which is secreted into the ECM, where it catalyzes formation of ε-(γ-glutamyl)lysine isopeptide, in a Ca^2+^-dependent manner.^16^ TG2 acts as an extracellular scaffold protein as well as a cross-linking enzyme. Numerous ECM proteins are TG2 substrates, such as fibronectin, collagen, and laminin.^16^ Under physiological conditions, TG2 regulates fibroblast activity and ECM organization, with little protein cross-linking. However, in pathologic conditions, increased TG2/ECM protein crosslinking and altered TG2 activity cause increased rigidity and stiffening of the vascular wall, processes that may contribute to remodelling in aging and cardiovascular disease. Recent evidence indicates altered TG2 activity and functionality in large arteries of hypertensive rats.^17^ TG2 dysregulation has also been implicated in small-vessel changes and inward remodelling in hypertension.^18^ Fundamental to many of the processes underlying ECM reorganization and fibrosis in aging and hypertension is activation of MMPs and TIMPs.

## MMPs and TIMPs

**Figure 2:**
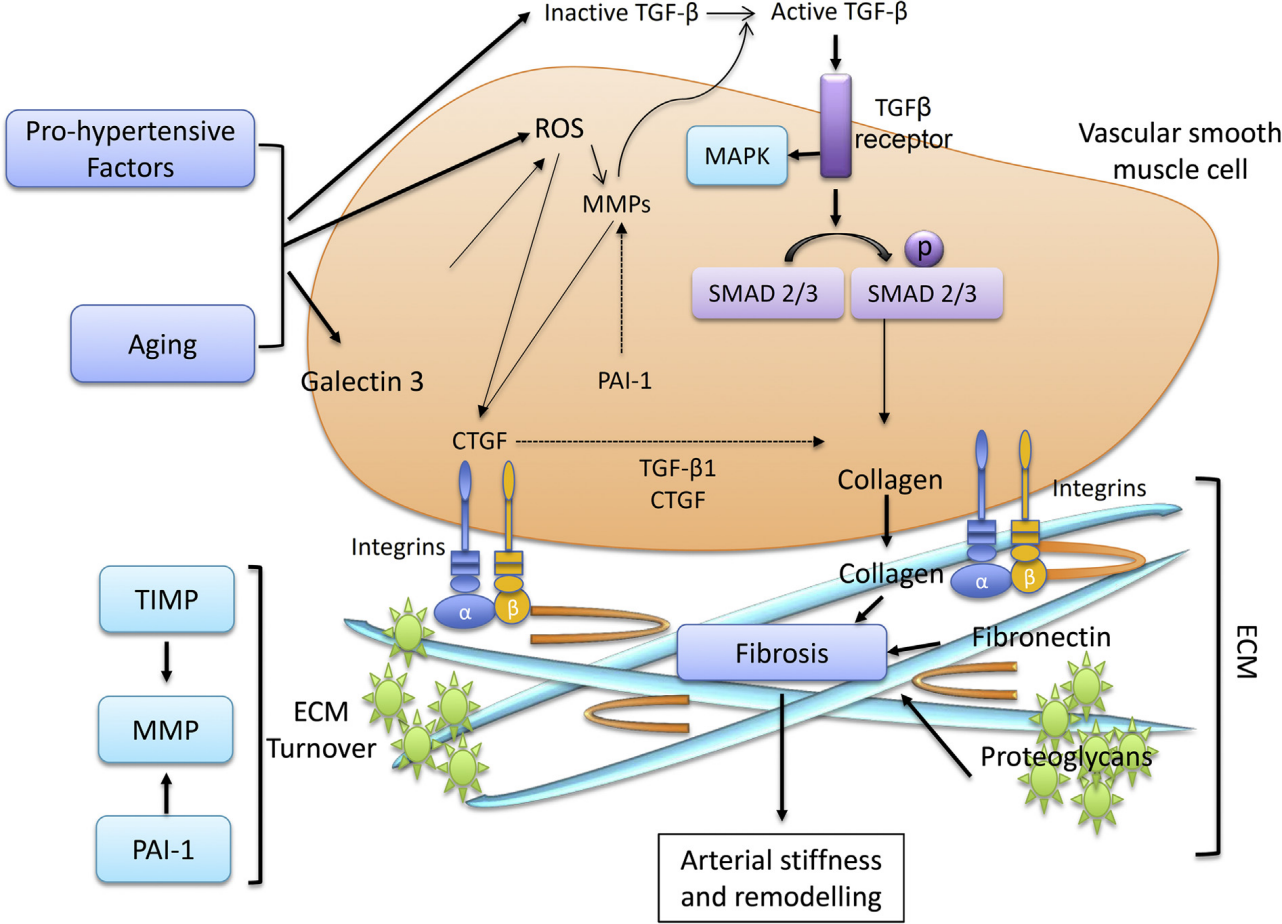
Vascular signalling mediating extracellular matrix (ECM) remodelling, fibrosis, and arterial stiffening in aging and hypertension. Prohypertensive factors and physiological aging promote ECM remodelling through activation of transforming growth factor-β (TGF-β) and subsequently, mitogen-activated protein kinase (MAPK) and SMAD pathways, reactive oxygen species (ROS) production, leading to matrix metalloproteinase (MMP) and connective tissue growth factor (CTGF) activation and upregulation of galectin-3. Subsequently, collagen, fibronectin, and proteoglycan deposition is increased, leading to fibrosis and increased arterial stiffness. PAI, plasminogen activator inhibitor.

ECM proteins, including collagen and elastin, are regulated by MMPs, a family of endopeptidases, which are activated by many factors associated with aging and hypertension, such as proinflammatory signalling molecules (cytokines, interleukins), growth factors, vasoactive agents (Ang II, ET-1, aldosterone) and ROS. MMP activity is controlled at 3 levels: gene transcription, proenzyme activation, and activity inhibition.^18^ Signalling pathways involved in regulating MMP transcription include p38 MAPK, which can enhance or repress MMP expression in a cell type–dependent manner (Fig. 2). Commonly, MMPs are activated in the pericellular space by other MMPs, including membrane-type MMPs and MMP-3, or by serine proteases like plasmin and chymase. Activated MMPs degrade collagen, elastin, and other ECM proteins, resulting in a modified ECM, often associated with a proinflammatory microenvironment that triggers a shift of endothelial and vascular smooth muscle cells to a more secretory, migratory, proliferative, and senescent phenotype, which contributes to fibrosis, calcification, endothelial dysfunction, and increased intima-media thickness, further impacting on vascular remodelling and arterial stiffness.

The effect that MMPs have on vascular fibrosis in hypertension is not completely elucidated, with both inhibitory and stimulatory modulation observed.^19^ This probably relates to activation of different MMP isoforms and downstream signalling pathways. For instance, MMP-1 overexpression attenuates fibrosis,^20^ whereas MMP-9 activation potentiates fibrosis and DNA damage.^21^ MMP2 activation leads to stimulation of TGF-β1 signalling; increased vascular smooth muscle cell production of collagens I, II, and III; and increased fibronectin secretion, processes that lead to collagen accumulation in the vascular wall. Although activation of vascular MMP2 and MMP9 in hypertension is associated with collagen accumulation, activation of MMP8 and MMP13 is associated with collagen degradation, processes especially important in arterial wall plaque and plaque rupture.^22^, ^23^ MMP2/MMP9 activation through TGF-β1/SMAD signalling also induces activation of myofibroblasts and increased infiltration of monocytes/macrophages, leading to oxidative stress, inflammation, and vascular wall injury. Vascular MMP2 and MMP9 are activated by numerous prohypertensive factors, including Ang II, ET-1, and salt, as well as mechanical and physical factors, such as shear stress and pressure. MMP2, MMP7, MMP9, and MMP14 are upregulated by aging. MMP2 activation is increased in aged rat aorta, leading to increased TGF-β1 and SMAD activation.^24^ Young rats infused with Ang II exhibit increased MMP2 activation with intima-media thickness and vascular fibrosis changes that are typical in old untreated rats.^24^ The importance of MMPs in vascular fibrosis in aging and hypertension is further evidenced by MMP inhibitors, such as PD166793, which blunted age-associated vascular fibrosis and remodelling in experimental models.^25^, ^26^

MMPs are normally inhibited by endogenous inhibitors called TIMPs, of which there are multiple isoforms. Alterations in the balance between ECM MMPs and TIMPs may contribute to the profibrotic phenotype in aging and hypertension.^19^, ^24^ The 4 TIMP isoforms—TIMP1, TIMP2, TIMP3, and TIMP4—are responsible for the inhibition of > 20 MMPs, and the relationship between MMPs and TIMPs changes with age. For instance, increased MMP2 expression and activity is observed in vessels of old rats and nonhuman primates compared with young counterparts.^5^, ^27^ Furthermore, TIMPs are downregulated in aged animals with heart failure but not in young animals.^28^

## Molecular and Cellular Mechanisms of Vascular Fibrosis in Aging and Hypertension

### TGF-β/SMAD signalling

The TGF-β superfamily consists of > 40 members that share common sequence elements and structural motifs and includes TGF-β, bone morphogenetic protein, activin, inhibin, and growth differentiation factors.^29^, ^30^, ^31^, ^32^ Disruption of the TGF-β pathway has been implicated in arterial aging and vascular fibrosis.^29^, ^30^, ^31^, ^32^ Three isoforms (TGF-β1, TGF-β2, and TGF-β3) exist; TGF-β1 is most frequently upregulated in ECM remodelling and fibrosis and is consequently regarded as an important regulator of the ECM. In the vascular system, TGF-β1 is expressed in endothelial cells, vascular smooth muscle cells, myofibroblasts, and adventitial macrophages. Activation of vascular TGF-β1, and its downstream signalling effector SMAD, increases the synthesis of ECM proteins such as fibronectin, collagen, and plasminogen activator inhibitor-1 (PAI-1).^33^, ^34^ TGF-β reduces collagenase production and stimulates expression of TIMPS, resulting in excessive matrix accumulation, in part resulting from inhibition of ECM degradation.^35^ TGF-β signalling predominantly occurs through the cytoplasmic proteins, SMADs, which translocate to the nucleus and act as transcription factors. The SMAD family comprises receptor-activated SMADs (SMAD2, SMAD3, SMAD5, and SMAD8), inhibitory SMADs (SMAD6, SMAD 7) and common-partner SMADs (SMAD4). SMAD2 and SMAD3 are specific mediators of TGFβ/activin pathways, whereas SMAD7 inhibits both BMP and TGF-β/activin signalling. SMAD activation results in increased transcription of many genes involved in ECM formation, including fibronectin, procollagens, PAI-1, and connective tissue growth factor (CTGF).^32^ In vascular smooth muscle cells, overexpression of SMAD7 inhibits TGF-β–induced fibronectin, collagen, and CTGF production.^36^ Important non-SMAD pathways implicated in TGF-β profibrotic signalling include extracellular signal-regulated kinase (ERK), c-Jun N-terminal kinase (JNK), p38 MAPK, and phosphoinositide 3-kinase/Akt.^37^ SMAD translocation to the nucleus can be modulated by Ras-activated ERK1/2. ERK inhibition reduces TGF-β–stimulated SMAD phosphorylation as well as collagen production, suggesting that ERK activation is necessary for an optimal response to TGF-β1.^36^

Activation of TGF-β1 and receptor-mediated signalling are increased in the aortic wall with aging and during development of hypertension.^24^ Important in the context of these conditions, Ang II,^38^, ^39^ mechanical stress,^34^, ^40^ ET-1,^36^and ROS^41^ are all elevated and are known to mediate TGF-β activation, with resultant vascular fibrosis. Additionally, MMPs (particularly MMP2 and MMP9) enhance release of TGF-β1, whereas TGF-β1 stimulates TIMP, resulting in inhibition of ECM degradation, which further induces ECM accumulation and vascular remodelling and fibrosis. Ang II can activate the SMAD pathway independent of TGF-β1, with implications for fibrosis.^36^, ^42^

### Plasminogen activator inhibitor-1

Plasminogen activator inhibitor-1 (PAI-1) is a member of the serine protease inhibitor (serpin) gene family and functions as an inhibitor of the serine proteases, urokinase-type plasminogen activator (uPA), and tissue-type plasminogen activator (tPA). PAI-1 inhibits fibrinolysis and hence regulates dissolution of fibrin and inhibits degradation of the ECM by reducing plasmin generation. PAI-1 normally maintains tissue homeostasis through regulating the activities of uPA, tPA, plasmin, and MMPs. In pathophysiological conditions, PAI-1 upregulation contributes to accumulation of ECM proteins and tissue fibrosis by preventing tissue proteolytic activity and reducing collagen degradation. Together with increased TGF-β1 activity, PAI-1 activity and expression are increased in experimental models of aging and in aged individuals.^43^, ^44^ PAI-1 is upregulated in aging-associated pathologic conditions, including hypertension.^45^ Increased PAI-1 is also recognized as a biomarker of cellular senescence in aging and hypertension.^46^

### Connective tissue growth factor

CTGF is a 38-kDa cysteine-rich secreted potent profibrotic factor implicated in fibroblast proliferation, cellular adhesion, and ECM synthesis. CTGF expression in the vasculature is enhanced by several stimuli, including TGF-β1, tumor necrosis factor-α, and mechanical stress.^47^ Ang II–induced vascular fibrosis is mediated by CTGF, and vascular smooth muscle cells treated with CTGF antisense oligonucleotides are protected against agonist-induced ECM protein expression.^36^, ^48^ CTGF may play an important role in arterial aging and vascular fibrosis; a number of experimental models have demonstrated increased levels of CTGF and associated vascular fibrosis with increasing age.^49^, ^50^

### Galectin-3

Galectin-3 (*LGALS3*) is a 29- to 35-kDa carbohydrate-binding lectin expressed on the cell surface of many cell types, including fibroblasts and endothelial and inflammatory cells. It is secreted mainly by activated macrophages, and it is ligand activated by oligosaccharides. Galectin-3 is also activated by other ligands, including glycosylated matrix proteins such as laminin, collagen, elastin, fibronectin, and integrin. The cellular actions of galectin-3 lead to cell proliferation, adhesion, and fibrosis. Galectin-3 has been shown to play an important role in fibrosis and tissue remodelling. In heart failure, plasma galectin-3 levels are increased.^51^ In the recent **Pre**vention of Renal and **V**ascular **End**-Stage Disease (PREVEND) study in which plasma galectin-3 levels were measured in 7968 individuals, plasma levels correlated positively with increasing age and cardiovascular risk factors, including hypertension.^52^ Because of its role in fibrosis, galectin-3 is now considered by many to be an important biomarker of cardiovascular fibrosis. The precise mechanisms through which galectin-3 influences ECM remodelling and fibrosis are still unclear, although activation of the Janus kinase (JAK)/signal transducer and activator of transcription (STAT) and protein kinase C (PKC) pathways,^53^, ^54^ as well as oxidative stress and inflammation, have been suggested. In addition, galectin-3 may directly increase production of ECM proteins. In rat vascular smooth muscle cells, overexpression of galectin-3 enhanced aldosterone-induced collagen 1 synthesis, whereas spironolactone or modified citrus pectin (galectin-3 inhibitor) reversed these effects.^55^ Galectin-3 inhibition also attenuated cardiovascular fibrosis and left ventricular dysfunction in a mouse model of heart failure.^56^

## The Role of Prohypertensive Vasoactive Factors in Vascular Aging and Fibrosis

**Figure 3:**
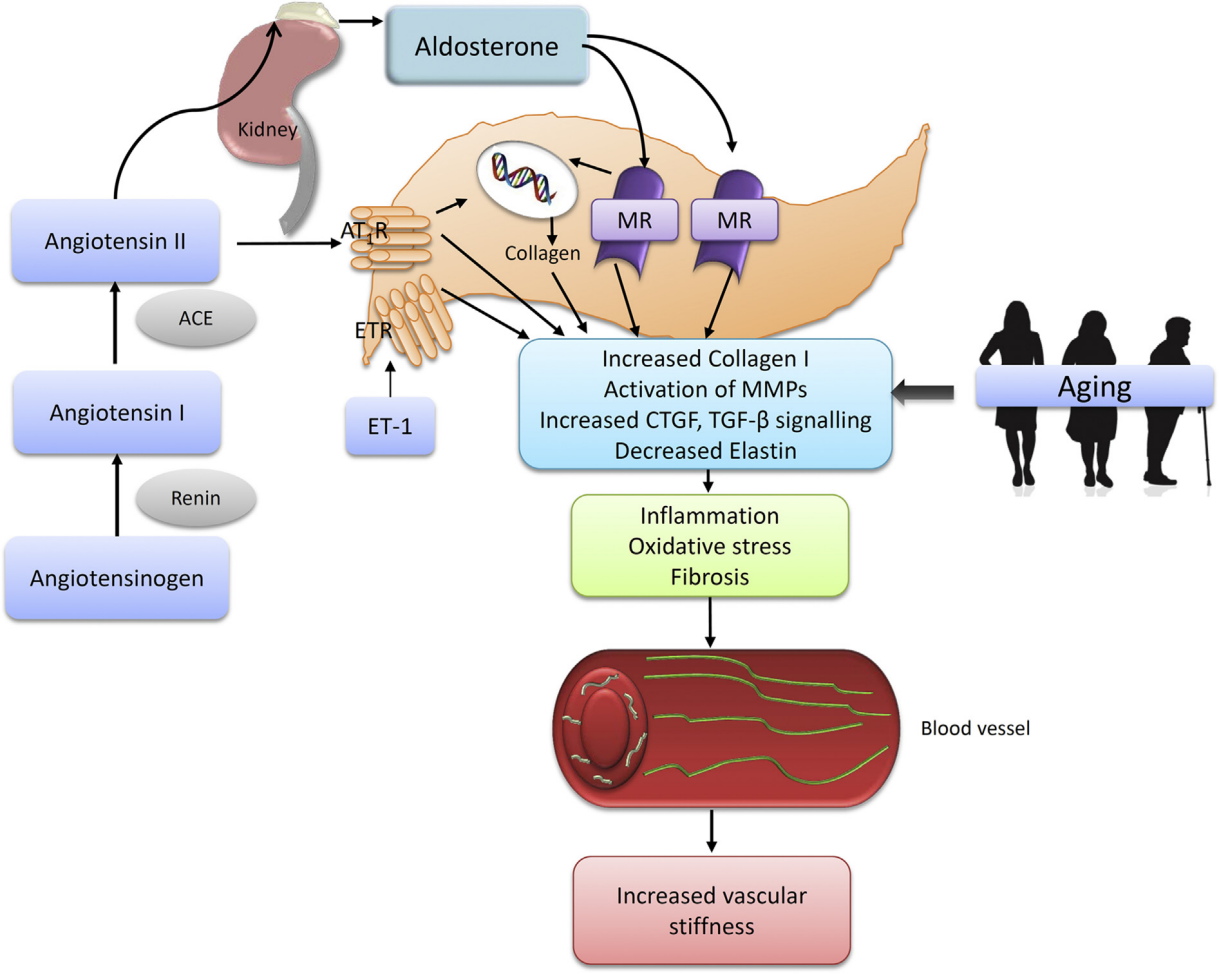
Influence of prohypertensive factors and aging in the development of vascular fibrosis and arterial stiffening. The renin-angiotensin-aldosterone system, acting through angiotensin receptor type 1 (AT1R) and mineralocorticoid receptor (MR), and endothelin-1 (ET-1) acting through endothelin receptor (ETR) activate matrix metalloproteinase (MMPs), connective tissue growth factor (CTGF), and transforming growth factor-β (TGF-β) signalling, resulting in inflammation, oxidative stress, and fibrosis, leading to increased arterial stiffness. This process is also induced by ET-1 signalling through ETR, aldosterone signalling through MR, and aging. ACE, angiotensin converting enzyme.

Many vasoactive factors activate profibrotic pathways, including Ang II, ET-1, and aldosterone (Figs. 2 and 3). Downstream signalling involves activation of redox-sensitive genes and transcription factors, early growth response factor-1, and activation of TGF-β1, MMPs, galectin-3, and MAP kinases.^57^, ^58^, ^59^, ^60^, ^61^ The aging vasculature is characterized by increased levels of Ang II,^5^ angiotensin-converting enzyme,^17^, ^31^, ^61^ mineralocorticoid receptors,^62^ and endothelin-converting enzyme-1.^63^, ^64^ As such, increased levels of these factors, their receptors, and downstream targets could represent an important event during aging that leads to vascular stiffness.

### Ang II signalling and vascular fibrosis

The renin-angiotensin-aldosterone system plays a central role in structural and mechanical changes in the vasculature. Ang II acts through activation of 2 receptors—AT1 and AT2_—_in which AT1 plays a major role in the production of ECM proteins.^65^, ^66^, ^67^, ^68^ This is highlighted by studies demonstrating that antagonism of Ang II receptors results in decreased fibrosis.^69^, ^70^ The precise signalling events involved in Ang II-induced vascular fibrosis are incompletely determined; however, in mesangial cells, TGF-β1 activity is increased by Ang II, an effect not observed when activator protein 1 binding sites or PKC- and p38 MAPK–dependent pathways are inhibited.^65^ In addition, galectin-3 seems to be associated with Ang II–induced fibrosis, and its expression is related to the severity of renal dysfunction in aging; mice subjected to Ang II infusion develop cardiac fibrosis,^71^ an effect not observed in galectin-3 knockout animals. Furthermore, cultured fibroblasts exposed to galectin-3 have reduced collagen production and deposition.^60^ Ang II–induced activation of p38 MAPK is also associated with the development and progression of fibrosis, commonly observed in aging and hypertension.^72^, ^73^, ^74^ It has been suggested that Ang II induces activity of MMPs and TIMPs and upregulation of CTGF during aging.^75^, ^76^, ^77^, ^78^, ^79^, ^80^

### Aldosterone and vascular fibrosis

Accumulating evidence implicates aldosterone as an important pathophysiological mediator in cardiovascular remodelling by promoting vascular hypertrophy, fibrosis, inflammation, and oxidative stress.^81^, ^82^, ^83^ Evidence from animal models and clinical trials of heart failure and hypertension demonstrate that chronic blockade of mineralocorticoid receptors, through which aldosterone signals, reduces cardiovascular fibrosis. In rats, aldosterone infusion increases aortic media cross-sectional area associated with elevated collagen levels, particularly increased collagen I synthesis.^84^, ^85^

In the context of aging, aldosterone levels have been shown to decline in older age.^86^, ^87^ This is associated with increased expression of mineralocorticoid receptors in intact vessels, as well as in cultured vascular smooth muscle cells, and has been shown to correlate with markers of vascular fibrosis.^62^ Whether increased signalling through mineralocorticoid receptors plays a role in vascular fibrosis associated with aging has yet to be confirmed.

### ET-1 and vascular fibrosis

ET-1 is a secreted peptide produced primarily in endothelial cells after conversion of preproendothelin to proendothelin and subsequently to mature endothelin, which has potent vasoconstrictor activity. The vascular actions of ET-1 are mediated by 2 distinct endothelin receptor subtypes: the ETA and ETB receptors located on both vascular smooth muscle and endothelial cells. In addition to well-established hypertrophic and mitogenic properties, ET-1 can modulate ECM remodelling by stimulating fibroblast-induced collagen synthesis. ET-1 stimulates synthesis of collagen through both ETA and ETB receptor subtypes.^88^, ^89^ Reduced cardiac and renal MMP activity and expression has been reported after administration of ETA receptor antagonists.^90^, ^91^, ^92^ Similarly, treatment with an endothelin antagonist normalizes expression of the collagen I gene and leads to the regression of renal vascular fibrosis and improved survival.^93^

Numerous findings have reported elevated ET-1 levels in healthy older adult humans.^94^, ^95^ In cultured aortic endothelial cells, ET-1 synthesis is greater in cells obtained from older donors vs young adult donors.^96^ In Wistar-Kyoto (WKY) rats, aging is associated with a 3.6-fold elevation in kidney ET-1 protein expression in the kidney. In rodent models, dual ETA/ETB receptor antagonism had no effect on the age-associated increase in aortic MMP-2 activity in WKY rats but markedly reduced pro and active MMP-2 activity in aged hypertensive rats, demonstrating that ET-1 may represent an important mediator of vascular stiffness in aging in the presence of other vascular diseases.^63^

## Conclusions

With aging, the vasculature undergoes structural and functional changes characterized by arterial remodelling, vascular fibrosis, and stiffening, which are processes that are evident in aging and hypertension. Arterial stiffening is common, occurring in > 60% of individuals older than 70 years and is a major independent predictor for serious cardiovascular events. Accordingly, there is a need to understand the fundamental processes that cause vascular stiffness so that mechanism-based therapeutic strategies can be developed to ameliorate or prevent processes of “vascular aging” in hypertension and associated cardiovascular diseases. Arterial stiffening is caused primarily by excessive fibrosis from excessive accumulation of vascular collagen and degradation of elastin. It is a dynamic phenomenon, which initially is an adaptive repair response that is reversible. However, the fibrogenic process is progressive, leading to further worsening of arterial stiffness and fibrosis that gradually extends into the neighbouring interstitial space, causing tissue and organ damage. A number of noninvasive methods are currently available to evaluate large-artery stiffness in the clinical setting, including carotid-femoral PWV. Increased PWV in aging and hypertension reflects increased arterial stiffness and is emerging as a biomarker for cardiovascular risk stratification. Perhaps over the next decade, PWV assessment may become a routine investigation in the clinical tool kit to better predict hypertension and cardiovascular disease.

## Funding Sources

This work was supported by grants from the British Heart Foundation (BHF) (RG/13/7/30099). R.M.T. is supported through a BHF Chair (CH/12/4/29762) and R.A.L. is supported by a PhD scholarship from FAPESP-Brazil (2012/12178-6).

## Disclosures

The authors have no conflicts of interest to disclose.
